# Palladium and Platinum Nanoparticles Attenuate Aging-Like Skin Atrophy via Antioxidant Activity in Mice

**DOI:** 10.1371/journal.pone.0109288

**Published:** 2014-10-15

**Authors:** Shuichi Shibuya, Yusuke Ozawa, Kenji Watanabe, Naotaka Izuo, Toshihiko Toda, Koutaro Yokote, Takahiko Shimizu

**Affiliations:** 1 Department of Advanced Aging Medicine, Chiba University Graduate School of Medicine, Chiba, Japan; 2 Department of Clinical Cell Biology and Medicine, Chiba University Graduate School of Medicine, Chiba, Japan; 3 Molecular Gerontology, Tokyo Metropolitan Institute of Gerontology, Itabashi, Tokyo, Japan; Oregon Health & Science University, United States of America

## Abstract

Cu-Zn superoxide dismutase (*Sod1*) loss causes a redox imbalance as it leads to excess superoxide generation, which results in the appearance of various aging-related phenotypes, including skin atrophy. Noble metal nanoparticles, such as palladium (Pd) and platinum (Pt) nanoparticles, are considered to function as antioxidants due to their strong catalytic activity. In Japan, a mixture of Pd and Pt nanoparticles called PAPLAL has been used to treat chronic diseases over the past 60 years. In the present study, we investigated the protective effects of PAPLAL against aging-related skin pathologies in mice. Transdermal PAPLAL treatment reversed skin thinning associated with increased lipid peroxidation in *Sod1* ^−/−^ mice. Furthermore, PAPLAL normalized the gene expression levels of *Col1a1*, *Mmp2*, *Has2*, *Tnf-α*, *Il-6*, and *p53* in the skin of the *Sod1* ^−/−^ mice. Pt nanoparticles exhibited marked SOD and catalase activity, while Pd nanoparticles only displayed weak SOD and catalase activity *in vitro*. Although the SOD and catalase activity of the Pt nanoparticles significantly declined after they had been oxidized in air, a mixture of Pd and Pt nanoparticles continued to exhibit SOD and catalase activity after oxidation. Importantly, a mixture of Pd and Pt nanoparticles with a molar ratio of 3 or 4 to 1 continued to exhibit SOD and catalase activity after oxidation, indicating that Pd nanoparticles prevent the oxidative deterioration of Pt nanoparticles. These findings indicate that PAPLAL stably suppresses intrinsic superoxide generation both *in vivo* and *in vitro* via SOD and catalase activity. PAPLAL is a potentially powerful tool for the treatment of aging-related skin diseases caused by oxidative damage.

## Introduction

Skin aging induced by chronological or intrinsic factors leads to skin atrophy [1]. The amounts of skin collagen components fall in an age-dependent manner in both males and females, resulting in age-related skin thinning in older individuals [2], [3]. Evidence suggests that oxidatively modified proteins, DNA, and lipids in the skin and other organs progressively accumulate during aging [4], indicating that reactive oxygen species (ROS) are strongly associated with skin aging. Complex organisms possess multiple antioxidative and repair systems for mitigating oxidative damage. Superoxide dismutase (SOD) plays a central role in antioxidative systems due to its ability to catalyze the conversion of cellular superoxide (O_2^−^_) to hydrogen peroxide (H_2_O_2_). H_2_O_2_ is further degraded to O_2_ and H_2_O by catalase, glutathione peroxidases and peroxiredoxins. Copper/zinc superoxide dismutase (SOD1) is localized to react with intracellular O_2_ in the cytoplasm. Our previous studies have demonstrated that *Sod1*-deficient (*Sod1* ^−/−^) mice exhibit increased intracellular O_2^−^_ concentrations and various aging-related organ phenotypes, such as age-related macular degeneration [5], fatty deposits in the liver [6], [7], skin atrophy [8]–[11], bone loss and fragility [12], [13], progression of Alzheimer's disease [14], infertility [15], dry eye [16], [17], and rotator cuff degeneration [18]. These findings suggest that cytoplasmic O_2^−^_-mediated oxidative damage is the primary cause of aging-related changes in various tissues. In particular, *Sod1* insufficiency results in both epidermal and dermal atrophy, which is associated with the downregulation of extracellular matrix related-genes, including *Col1a1* and *Has2* [11]. Therefore, *Sod1* ^−/−^ mice constitute a suitable model for studying skin aging in elderly people.

Noble metal nanoparticles, including those palladium (Pd), platinum (Pt), and gold nanoparticles, display strong catalytic activity, e.g., in hydrogenation, hydration, and oxidation reactions, due to their large surface area and the high proportion of metal atoms located on their surfaces [19]–[21]. Such noble metal nanoparticle catalysts are considered to function as antioxidants and are potentially useful in material science and engineering as well as in medical science and clinical therapy [22]. A number of studies have reported that Pt nanoparticles exhibit strong antioxidant activity [22]–[24]. Recently, Elhusseiny and Hassan reported that a complex of Pd and Pt nanoparticles demonstrated highly potent antitumor and antimicrobial activity [25]. In 1915, Dr. Hideyo Noguchi (Rockefeller University) and Dr. Saburo Ishizuka (a dental surgeon at Tokyo Dental College) formulated a plan for creating a solution of Pd and Pt nanoparticles for clinical use. Twenty-one years later, Dr. Ishizuka successfully prepared a Pd and Pt nanoparticle solution called PAPLAL (Toyokose Pharmaceuticals, Japan) [26]. Since then, PAPLAL has been used to treat Japanese patients with burns, frostbite, hives, lung inflammation, gastric ulcers, and rheumatoid arthritis. PAPLAL has been shown to have various beneficial effects on chronic diseases [26]. In addition, PAPLAL has been approved as a treatment for acute gastric inflammation and chronic gastrin catarrh in Japan under the Pharmaceutical Affairs Law, and it has also been patented as an antioxidant with the Japan Patent Office (Patent No. 3411195, 2003). *In vitro* studies have reported that PAPLAL exhibits antioxidant activity against superoxide anions and hydroxyl radicals [27], [28]. However, no previous studies have investigated the effects of PAPLAL or other metal nanoparticles on skin aging.

In the present study, we investigated the protective effects of PAPLAL against age-related skin pathologies in model mice. We also analyzed the expression profiles of skin-related genes, including those involved in matrix biosynthesis, inflammation, and aging, in order to clarify the underlying mechanisms of the i*n vivo* effects of PAPLAL. In addition, *in vitro* experiments were conducted to evaluate the antioxidant activity of PAPLAL.

## Materials and Methods

### Nanoparticles

Pd and Pt nanoparticles and PAPLAL were provided by Toyokose Pharmaceutical Co. (Tokyo, Japan) through Musashino Pharmaceutical Co. (Tokyo Japan). PAPLAL is composed of a mixture of 0.3 mg/mL (2.82 mM) of Pd nanoparticles and 0.2 mg/mL (1.03 mM) of Pt nanoparticles.

### Mice

*Sod1* ^−/−^ mice were purchased from the Jackson Laboratory (Bar Harbor, ME, USA). Genotyping of the *Sod1* ^−/−^ allele was performed via genomic PCR using genomic DNA isolated from the tail tip, as reported previously [8]. The animals were housed under a 12-hour light/dark cycle and fed *ad libitum*. In addition, they were maintained and studied according to protocols approved by the animal care committee of the Tokyo Metropolitan Institute of Gerontology and Chiba University.

### Transdermal administration in *Sod1* ^−/−^ mice

The *Sod1* ^+/+^ and *Sod1* ^−/−^ mice were transdermally administered PAPLAL for four weeks, beginning at four months of age. On the first day of each week, the hair on the backs of the mice was shaved off and then PAPLAL (200 µL/mouse) or MilliQ water (200 µL/mouse) was applied to the exposed skin once a day. MilliQ water was used as a placebo control for PAPLAL.

### Histology

To assess the histological morphology of the treated skin, skin specimens were dissected from the back tissue of the mice, fixed overnight in a 20% formalin neutral buffer solution (Wako, Osaka, Japan), embedded in paraffin, and sectioned on a microtome at a thickness of 4 µm according to standard techniques. Hematoxylin and eosin staining was performed as described previously [6], [29]. The thickness of the skin tissue was measured using the Leica QWin V3 imaging software (Leica, Germany).

### 8-isoprostane content

The skin tissue specimens were homogenized with 0.1 M phosphate (pH 7.4) containing 1 mM of ethylenediaminetetraacetic acid (Dojindo Laboratories, Kumamoto, Japan) and 50 µg/mL (w/w) of dibutylhydroxytoluene (Wako). The homogenate was centrifuged at 8,000×*g* for 10 minutes at 4°C, and the total supernatant was used for the assay. The 8-isoprostane concentration of the homogenate was measured using an 8-isoprostane enzyme immunoassay kit (Cayman Chemical Company, MI, USA) according to the manufacturer's instructions. The protein concentration of the supernatant was assessed using the DC protein assay kit (BioRad, Hercules, CA, USA), and the 8-isoprostane level was normalized to the protein level.

### Outgrowth assay

Mouse back skin samples were sterilized with 70% ethanol and rinsed with phosphate-buffered saline (Takara Bio Inc., Shiga, Japan), and then discs measuring 5 mm in diameter were punched out using a dermal punch (Nipro, Tokyo, Japan). The punched skin discs were placed into a 24-well culture plate (Falcon BD, Franklin Lakes, NJ) and cultured with or without PAPLAL in α-minimum essential medium (α-MEM; Life Technologies Corporation, Carlsbad, CA, USA) containing 20% fetal bovine serum (FBS; Life Technologies Corporation), 100 units/mL of penicillin, and 0.1 mg/mL of streptomycin (Sigma-Aldrich, MO, USA) at 37°C in a humidified incubator under 5% CO_2_ and 20% O_2_. The number of outgrowing fibroblasts originating from the mouse skin discs was directly counted after 96 h culturing.

### Lactate dehydrogenase (LDH) activity

Skin specimens were cultured according to the method described above, and the culture medium was collected after 96 h. The collected medium was centrifuged at 400×*g* for 5 min at 4°C, and the total supernatant was used for the subsequent assay. The LDH level was measured using the LDH cytotoxicity assay kit (Cayman Chemical Company) according to the manufacturer's instructions.

### Quantitative PCR

Total RNA was extracted from the back skin using Trizol reagent (Life Technologies Corporation) according to the manufacturer's instructions. cDNA was synthesized from 1 µg of total RNA using reverse transcriptase (ReverTra Ace qPCR RT Master MIX, TOYOBO, Osaka, Japan). Real-time PCR was performed on a Mini Opticon™ (Bio-Rad) with SYBR GREEN PCR master mix (Bio-Rad), according to the manufacturer's instructions. All expression data were normalized to the expression level of the housekeeping gene glyceraldehyde-3-phosphate dehydrogenase (*Gapdh*). The primer sequences are listed in Table S1.

### Antioxidant activity

The SOD and catalase activity of the Pd and Pt nanoparticles was measured using a WST-based SOD assay kit (Dojindo Laboratories) according to the manufacturer's instructions, whereas the catalase activity of the Pd and Pt nanoparticles was evaluated using an Amplex Red catalase assay kit (Sigma-Aldrich) according to the manufacturer's instructions. SOD derived from bovine erythrocytes (Sigma-Aldrich, lot number 080M76901V) and catalase derived from bovine liver tissue (Sigma-Aldrich, lot number 1232075) were used as positive controls for SOD and catalase activity, respectively. In order to assess the stability of the antioxidant activity of the different nanoparticles, Pd and Pt nanoparticle solutions were incubated at room temperature for four weeks in tubes in which the air had been replaced with N_2_. In a further experiment, Pd and Pt nanoparticle solutions were exposed to air by rotating them for 24 h at room temperature. The SOD and catalase activity levels of each solution were then measured as described above.

### Statistics

Statistical analyses were performed using the Student's *t*-test for comparisons between two groups or Tukey's test for comparisons between three groups. Differences were considered significant at p-values of less than 0.05. All data are expressed as mean ± standard deviation (SD) values.

## Results

### PAPLAL accelerated wound healing in the aged mice

In order to evaluate the protective effects of PAPLAL on the skin, we first investigated the ability of PAPLAL to promote wound healing in aged murine skin (17 months of age). Although the areas of the wounds treated with and without PAPLAL did not differ at two days after wounding, the wounds treated with PAPLAL were significantly smaller than those treated with vehicle at four and six days after wounding (Figure S1A–1B). In addition, PAPLAL treatment was found to have promoted wound healing at 12 days after wounding (Figure S1B). These results demonstrate that the transdermal application of PAPLAL assists in wound healing in aged mice.

### PAPLAL attenuated skin atrophy in the *Sod1*-deficient mice

**Figure 1:**
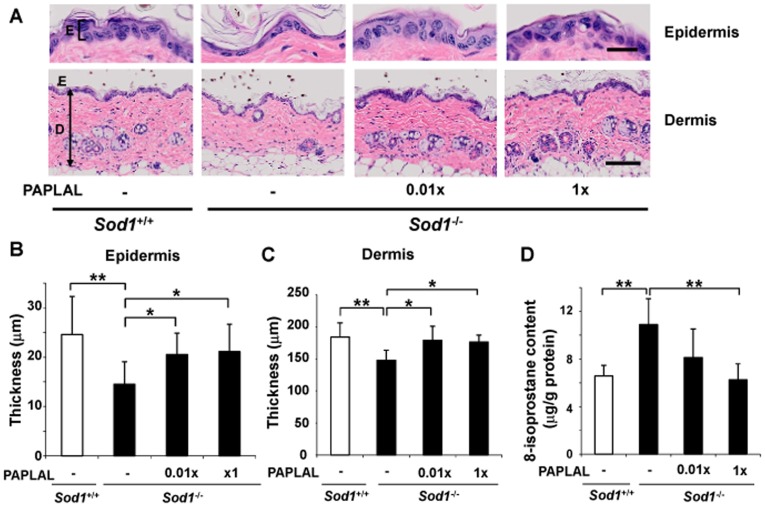
Protective effects of PAPLAL against skin atrophy in the *Sod1*-deficient mice. (A) Hematoxylin and eosin staining of the skin on the backs of the *Sod1* ^+/+^ and *Sod1* ^−/−^ mice (five months of age). E, epidermis; D, dermis. The scale bars represent 20 µm (top) or 100 µm (bottom). The thickness of the (B) epidermal and (C) dermal layers of the skin on the backs of *Sod1* ^+/+^ and *Sod1* ^−/−^ mice treated with PAPLAL (n = 6–8). (D) 8-isoprostane content of the skin on the backs of *Sod1* ^+/+^ and *Sod1* ^−/−^ mice treated with PAPLAL (n = 5–8). 0.01× and 1× PAPLAL indicate 0.01- or 1-fold concentrations of PAPLAL, respectively. Data are shown as the mean ± SD; *p<0.05, **p<0.01.

SOD1, an antioxidant enzyme, plays a pivotal role in cellular antioxidative systems. Therefore, in order to investigate the beneficial effects of PAPLAL on the skin symptoms seen in *Sod1* ^−/−^ mice, PAPLAL was transdermally administered to the skin on the backs of *Sod1* ^−/−^ and wild-type (*Sod1* ^+/+^) mice daily for four weeks, beginning at four months of age. As shown in Figure 1, the skin of the *Sod1* ^−/−^ mice was significantly thinner than that of the *Sod1* ^+/+^ mice, confirming that skin atrophy had occurred in the *Sod1* ^−/−^ mice. The back skin of the *Sod1* ^−/−^ mice that were treated with a high concentration of PAPLAL was significantly thicker (epidermis and dermis: by 46.2% and 19.2%, respectively) than that of the *Sod1* ^−/−^ mice treated with the vehicle. The administration of a low concentration of PAPLAL also improved the skin atrophy observed in the *Sod1* ^−/−^ mice (the thickness of the epidermis and dermis were increased by 42.0% and 21.1%, respectively) compared with that seen in the *Sod1* ^−/−^ mice treated with vehicle. In order to examine the degree of oxidative damage in the skin of the *Sod1* ^−/−^ mice, we measured the concentration of 8-isoprostane as a representative of lipid peroxidation products. The 8-isoprostane content of the *Sod1* ^−/−^ skin was increased by 65.4% compared with that of the *Sod1* ^+/+^ mice, which indicated that lipid peroxidation products had accumulated in it (Figure 1D). Meanwhile, treatment with a high concentration of PAPLAL significantly decreased the 8-isoprostane content of the *Sod1* ^−/−^ mouse skin compared with that of the *Sod1* ^−/−^ mouse skin treated with the vehicle (Figure 1D).

**Figure 2:**
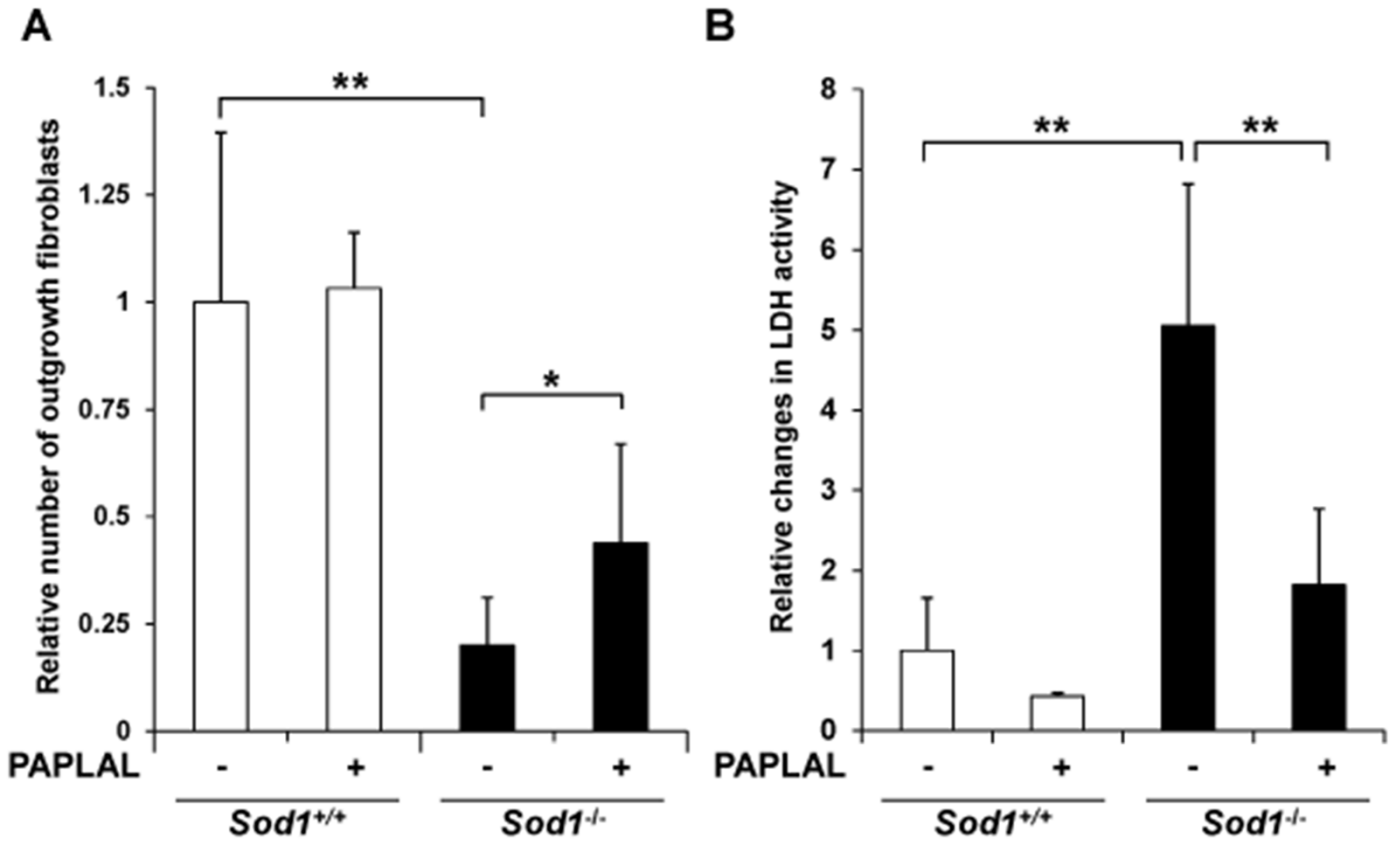
PAPLAL attenuates cellular damage in skin. (A) Relative number of outgrowing fibroblasts in cultured *Sod1* ^+/+^ and *Sod1* ^−/−^ skin specimens. (B) LDH activity in the medium used to culture the *Sod1* ^+/+^ and *Sod1* ^−/−^ skin specimens. Data are shown as the mean ± SD; *p<0.05, **p<0.01.

Next, we investigated the protective effects of PAPLAL against skin damage. Organ culture experiments using skin discs demonstrated that the *Sod1* ^−/−^ fibroblasts had a markedly lower outgrowth capacity than the *Sod1* ^+/+^ fibroblasts, which indicated that the migration and proliferation of the *Sod1* ^−/−^ fibroblasts were impaired (Figure 2A). In order to analyze the protective effects of PAPLAL, we added PAPLAL to cultures of *Sod1* ^−/−^ skin discs. Treatment with PAPLAL significantly promoted fibroblast outgrowth from the mutant skin discs (Figure 2A). We also measured the LDH activity, which is a marker of skin damage, in the culture medium. The *Sod1* ^−/−^ skin exhibited significantly increased LDH activity by 5.1-fold compared with the *Sod1* ^+/+^ fibroblasts, which was indicative of skin damage (Figure 2B). Meanwhile, PAPLAL treatment significantly suppressed the LDH activity in the *Sod1* ^−/−^ skin disc culture compared with vehicle treatment (Figure 2B).

**Figure 3:**
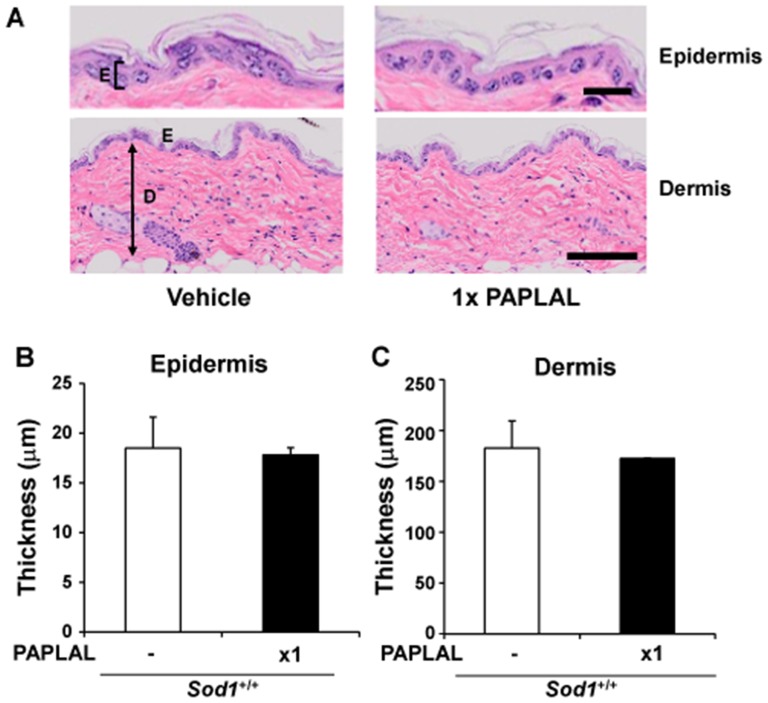
PAPLAL is non-toxic in wild-type mice. (A) Hematoxylin and eosin staining of the skin on the backs of *Sod1* ^+/+^ mice (17–20 weeks of age). E, epidermis; D, dermis. The scale bars represent 20 µm (top) or 100 µm (bottom). The thickness of the (B) epidermal and (C) dermal layers of the skin on the backs of *Sod1* ^+/+^ mice treated with PAPLAL (n = 5).

In order to investigate the adverse effects of PAPLAL, we transdermally administered PAPLAL into the skin on the backs of *Sod1* ^+/+^ mice. As shown in Figure 3, no significant differences in skin thickness were observed among the *Sod1* ^+/+^ mice treated with or without PAPLAL. In addition, no abnormalities, such as cell infiltration or PAPLAL deposition, were detected in the skin of the *Sod1* ^+/+^ mice, suggesting that PAPLAL does not have any adverse effects on the skin, at least in the short term. In the organ culture analysis, PAPLAL treatment of *Sod1* ^+/+^ skin did not induce any significant change in fibroblast outgrowth capacity or LDH activity detected in skin disc cultures (Figure 2A, 2B). These findings demonstrate that the transdermal application of PAPLAL ameliorates skin atrophy in *Sod1* ^−/−^ mice by suppressing oxidative damage.

### PAPLAL normalized the transcriptional profiles of skin-related genes in the *Sod1* ^−/−^ skin

**Figure 4:**
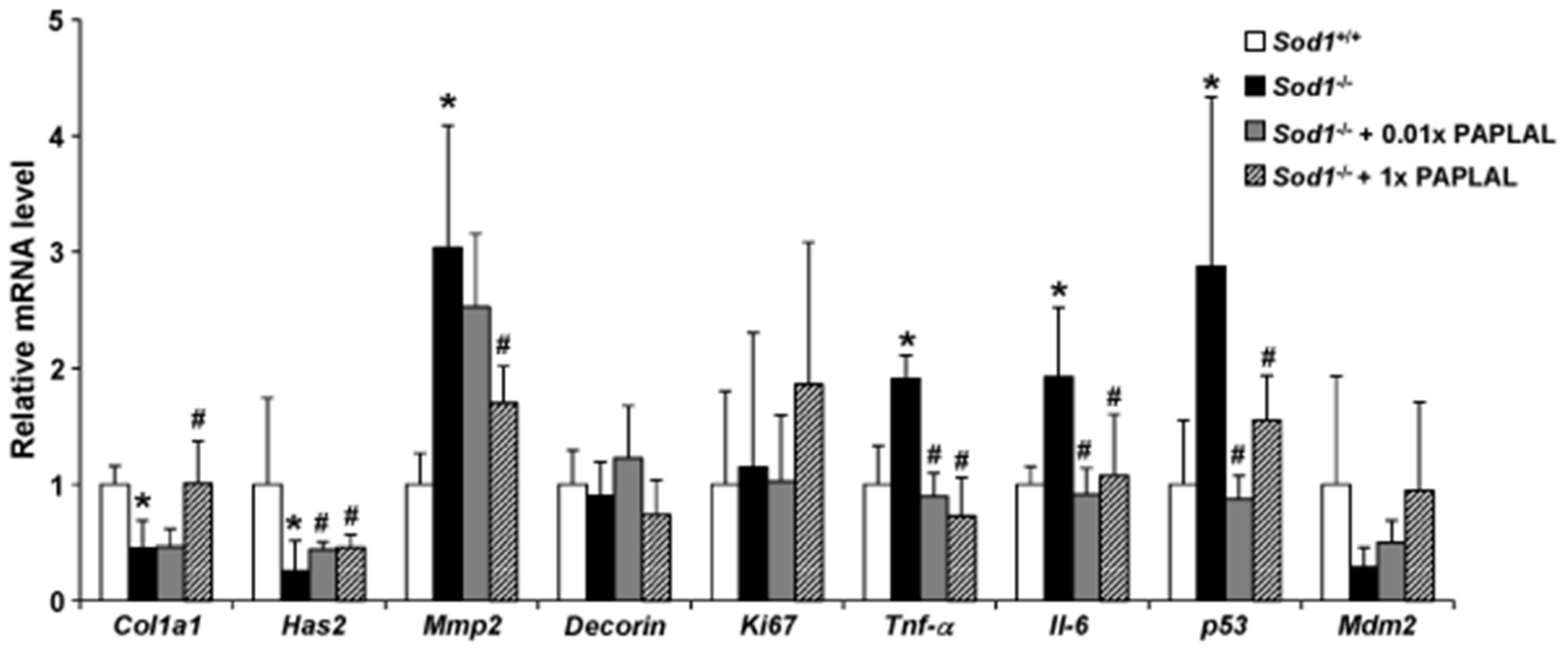
PAPLAL improved the transcriptional profiles of skin-related genes in the skin of *Sod1* ^−/−^ mice. (A) The relative mRNA expression levels of *Col1a1*, *Has2*, *Mmp2*, *Decorin*, *Ki67*, *Tnf-α*, *Il-6*, *p53*, and *Mdm2*. Each mRNA expression level was determined using qRT-PCR. Data are shown as the mean ± SD; *p<0.05 vs. *Sod1* ^+/+^, **p<0.01 vs. *Sod1* ^+/+^, ^#^ *p*<0.05 vs. *Sod1* ^−/−^.

In order to investigate the mechanisms by which PAPLAL treatment counters skin atrophy in *Sod1* ^−/−^ mice, we analyzed the expression patterns of extracellular matrix-related genes in the skin. In the *Sod1* ^−/−^ mice, the skin mRNA expression level of the type I collagen gene (*Col1a1*) was significantly reduced, while that of the matrix metalloproteinase 2 gene (*Mmp2*) were significantly increased, compared with those observed in the *Sod1* ^+/+^ mice, indicating that collagen biosynthesis was reduced in the *Sod1* ^−/−^ mice (Figure 4). Moreover, the mRNA expression of the hyaluronan synthase 2 gene (*Has2*) was significantly downregulated in the skin of the *Sod1* ^−/−^ mice (Figure 4). In contrast, the mRNA expression levels of *Decorin* and *Ki67* did not differ between the *Sod1* ^+/+^ and *Sod1* ^−/−^ mice (Figure 4). These results suggest that *Sod1* deficiency causes skin thinning due to dysregulation of the extracellular matrix. Among the genes that exhibited altered expression levels, PAPLAL treatment significantly normalized the mRNA levels of *Col1a1*, *Mmp2*, and *Has2*, suggesting that PAPLAL treatment increases skin thickness by increasing the concentrations of extracellular matrix components, such as collagen and hyaluronic acid (Figure 4).

The skin of the *Sod1* ^−/−^ mice also exhibited significantly higher expression levels of inflammatory cytokines, including *Tnf-*α and *Il-6*, compared with the skin from the *Sod1* ^+/+^ mice (Figure 4). PAPLAL significantly downregulated the mRNA expression levels of *Tnf-*α and *Il-6* in the skin of the *Sod1^−/−^* mice, suggesting that a pathological link exists between inflammation and skin thinning in *Sod1* ^−/−^ mice (Figure 4). Furthermore, the expression of the tumor suppresser *p53* gene, which is known to be associated with DNA damage [30] and skin aging [31], was significantly upregulated in the *Sod1* ^−/−^ mice (Figure 4). In contrast, *Mdm2* expression tended to be downregulated in the skin of the *Sod1^−/−^* mice. PAPLAL treatment significantly normalized the mRNA expression level of *p53* of the *Sod1^−/−^* mice (Figure 4), suggesting that PAPLAL delays skin aging by inhibiting p53 upregulation in *Sod1* ^−/−^ mice.

### Pd nanoparticles prevented the oxidative deterioration of Pt nanoparticles with respect to their SOD and catalase activity

**Figure 5:**
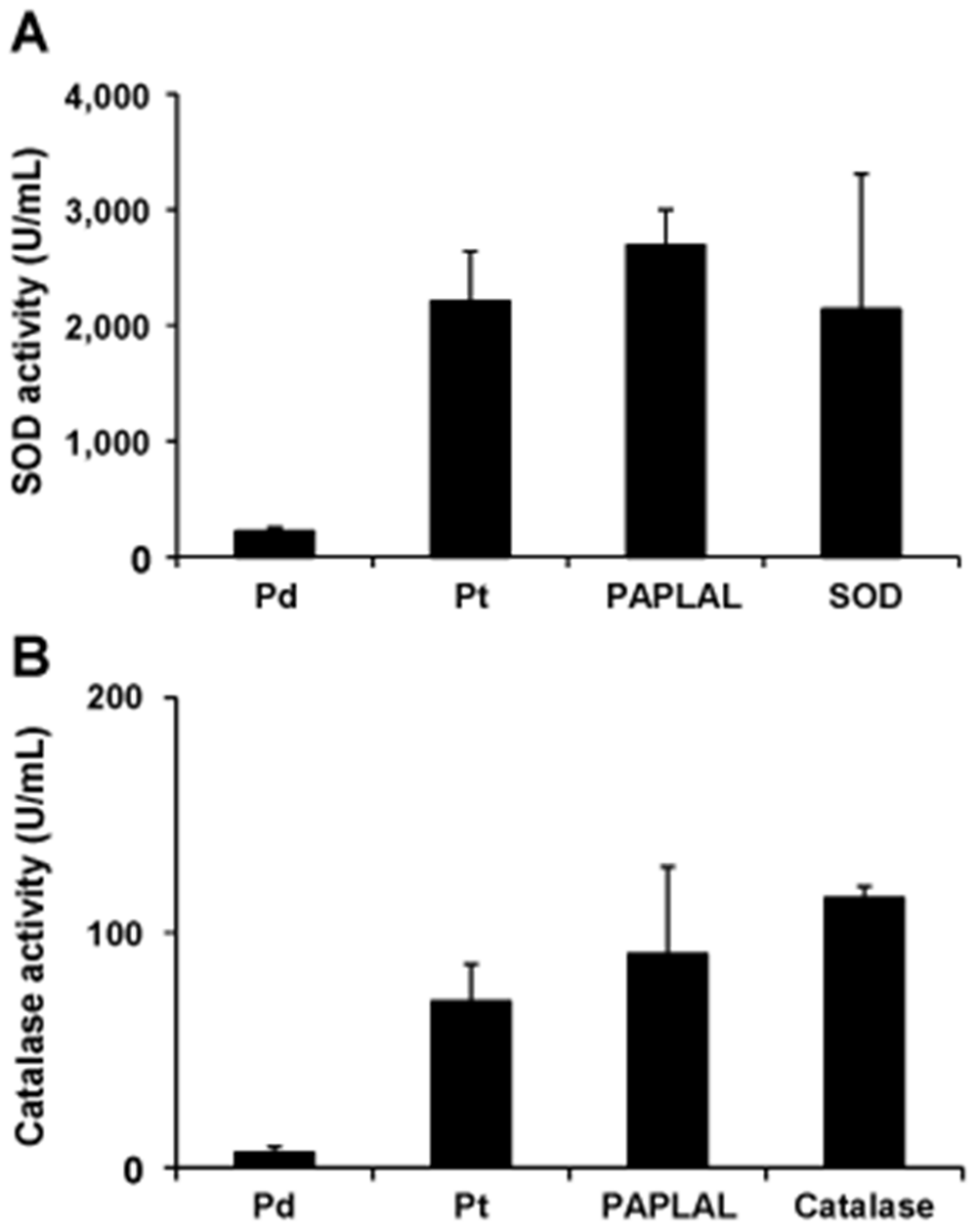
Pt nanoparticles possess SOD and catalase activity. PAPLAL includes 2.82 mM of Pd nanoparticles and 1.03 mM of Pt nanoparticles. (A) The SOD and (B) catalase activity of 2.82 mM Pd nanoparticles, 1.03 mM Pt nanoparticles, and PAPLAL. Five nM of SOD derived from bovine erythrocytes (A) and 0.2 µM of catalase derived from bovine liver tissue (B) were used as positive controls for SOD and catalase, respectively.

Since Pt nanoparticles display strong antioxidant activity [22]–[24], we assessed the SOD and catalase activity of PAPLAL and its components *in vitro*. The Pd nanoparticles displayed weak SOD and catalase activity, while the Pt nanoparticles exhibited strong SOD and catalase activity (Figure 5A, 5B). PAPLAL, a mixture of Pd and Pt nanoparticles, demonstrated SOD and catalase activity levels that were equivalent to the sum of those exhibited by the Pd and Pt nanoparticles, although no synergistic effects were observed (Figure 5A, 5B).

**Figure 6:**
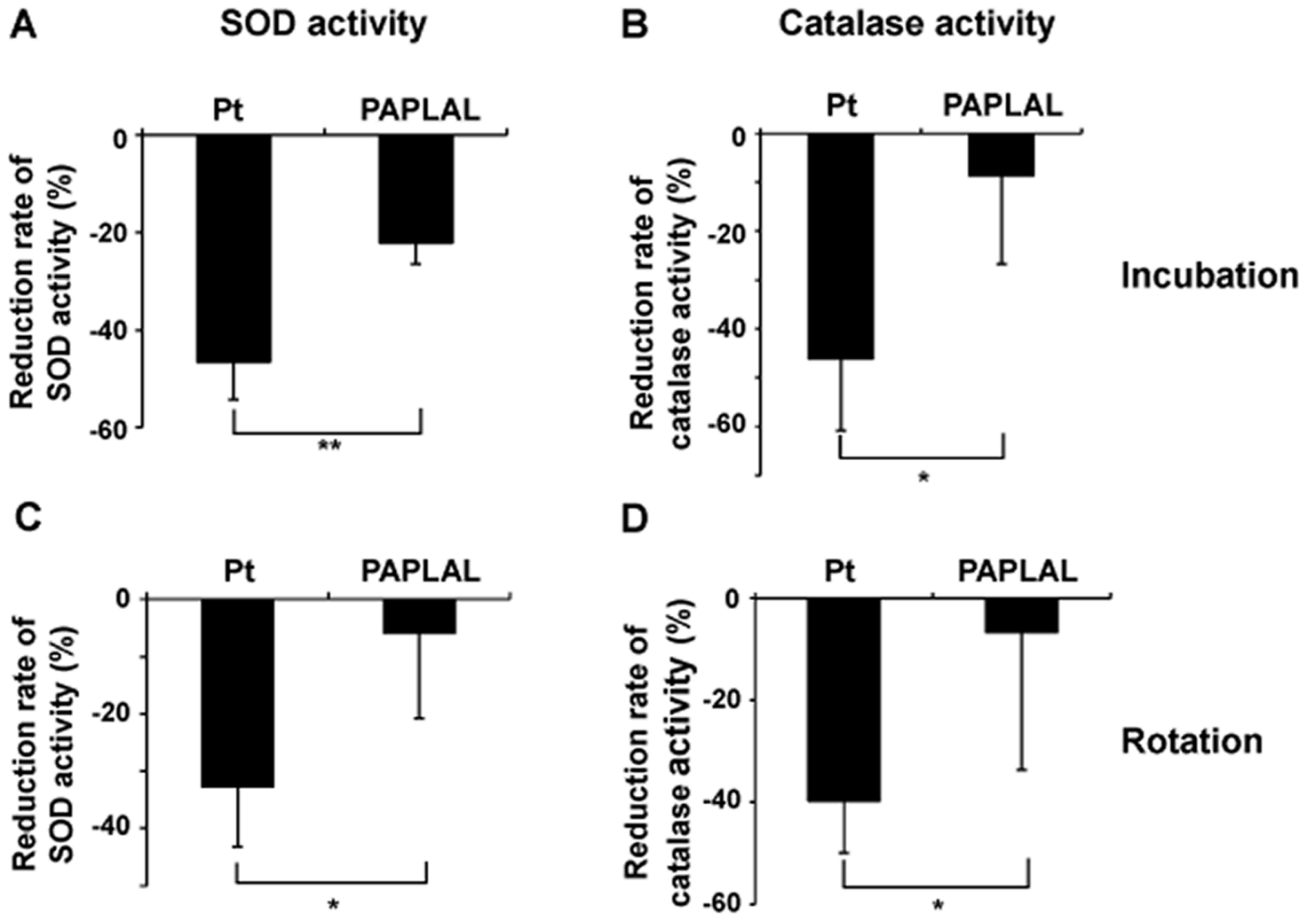
Pd nanoparticles protected the SOD and catalase activity of Pt nanoparticles against oxidative degradation *in vitro*. (A) The SOD and (B) catalase activity of 1.03 mM Pt nanoparticles and PAPLAL that had been stored at room temperature for four weeks. (C) The SOD and (D) catalase activity of Pt nanoparticles and PAPLAL that had been rotated for 24 hours in a tube in order to oxidize the nanoparticles. Data are shown as the mean ± SD; *p<0.05, **p<0.01.

In order to evaluate the stability of the SOD and catalase activity of the tested nanoparticles, Pd and Pt nanoparticles and PAPLAL were stored at room temperature for four weeks. The SOD and catalase activity levels of the stored Pt nanoparticles were dramatically reduced to 46.5% and 46.2%, respectively, of those exhibited by the freshly prepared Pt nanoparticles (Figure 6A, 6B). In contrast, the SOD and catalase activity levels of stored PAPLAL were only reduced by 22.1% and 8.7%, respectively, compared with those of the freshly prepared PAPAL (Figure 6A, 6B). In order to confirm that the reductions in the SOD and catalase activity of the Pt nanoparticles were due to oxidative deterioration during storage, Pt nanoparticles and PAPAL were exposed to air by rotating them for 24 hours. The SOD and catalase activity levels of the Pt nanoparticles that were exposed to air were significantly decreased compared with those of the freshly prepared nanoparticles (SOD and catalase: 33.0% and 39.7%, respectively; Figure 6C, 6D). In contrast, the PAPLAL exposed to air retained its SOD and catalase activity, indicating that the oxidative deterioration of the Pt nanoparticles within it had been prevented (Figure 6C, 6D).

**Figure 7:**
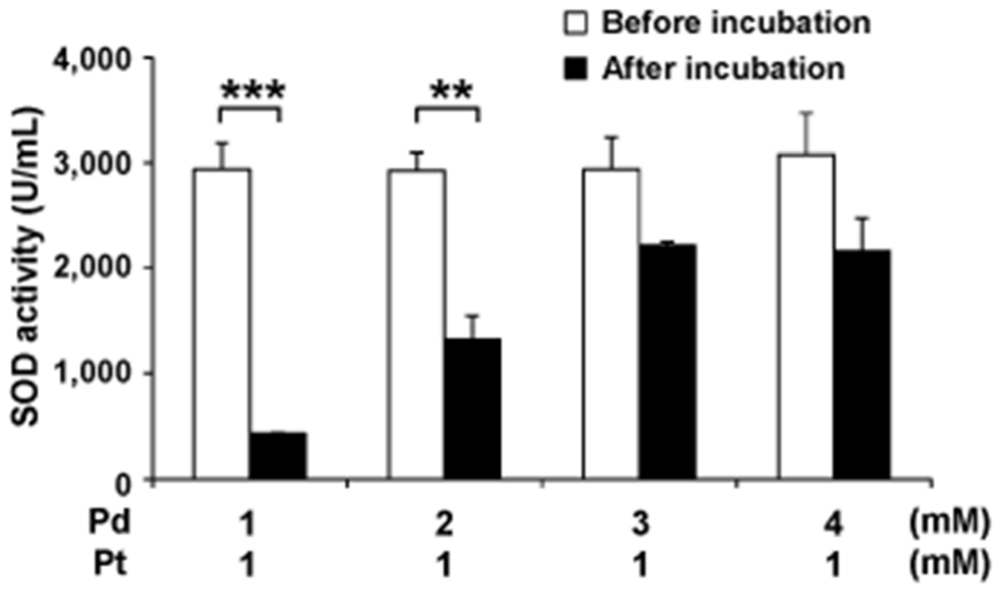
Pd nanoparticles protected the SOD activity of Pt nanoparticles against oxidative degradation at various molar ratios. Pd nanoparticles were added to Pt nanoparticles at various molar ratios, and the SOD activity of each mixture was measured after four weeks of storage at room temperature.

Furthermore, Pd nanoparticles were mixed with Pt nanoparticles at various concentration ratios and then stored at room temperature for four weeks. When Pd nanoparticles were added to Pt nanoparticles at a molar ratio of 1 or 2 to 1, the SOD and catalase activity levels of the mixture were markedly reduced after their storage at room temperature (Figure 7). However, when Pd nanoparticles were added to Pt nanoparticles at a molar ratio of 3 or 4 to 1, the SOD and catalase activity levels of the mixture were sustained (Figure 7).

We previously reported that *Sod1* loss significantly enhanced intracellular O_2^−^_ generation in fibroblasts [9], [32]. Therefore, we assessed the antioxidant effects of Pd and Pt nanoparticles on *Sod1* ^−/−^ fibroblasts. *Sod1* ^−/−^ fibroblasts were treated with Pd and/or Pt nanoparticles for 16 hours, and then the levels of intracellular O_2^−^_ were assessed. O_2^−^_ generation was significantly decreased (by 23.3%) in the *Sod1* ^−/−^ fibroblasts treated with 10 µM of Pt nanoparticles, but not in those treated with Pd nanoparticles (Figure S2). Notably, treatment with pre-incubated Pt nanoparticles did not decrease intracellular O_2^−^_ generation in the *Sod1* ^−/−^ fibroblasts (Figure S2). These results suggest that Pt nanoparticles possess strong antioxidant effects, such as SOD and catalase activity, and that the Pd in PALAL inhibits the oxidative deterioration of Pt, which enables PALAL to retain strong antioxidant activity over time.

## Discussion

### PAPLAL attenuates intrinsic skin aging by suppressing oxidative damage

In the present study, we demonstrated that PAPLAL significantly reversed skin thinning by reducing oxidative and cellular damage in *Sod1* ^−/−^ mice (Figures 1 and 2). In addition, PAPLAL and Pt nanoparticles, but not Pd nanoparticles, exhibited SOD and catalase activity (Figure 5). Furthermore, an *in vitro* experiment found that treatment with Pt nanoparticles, but not Pd nanoparticles, significantly reduced O_2^−^_ generation in *Sod1* ^−/−^ fibroblasts (Figure S2). These findings suggest that the antioxidant activity of the Pt nanoparticles in PAPLAL contribute to attenuating age-related skin thinning in *Sod1* ^−/−^ mice. In this context, we previously evaluated the ability of several antioxidants to counteract the *in vivo* age-related changes seen in *Sod1* ^−/−^ mice and found that the administration of ascorbic acid significantly attenuated bone loss and fragility in *Sod1* ^−/−^ mice [12]. Likewise, the transdermal administration of ascorbic acid derivatives was demonstrated to normalize skin thinning in *Sod1* ^−/−^ mice [9], [10]. Furthermore, Iuchi *et al.* reported that oral N-acetylcysteine treatment mitigates hemolytic anemia in *Sod1* ^−/−^ mice by suppressing ROS generation in red blood cells [33]. Together, these findings demonstrate that antioxidants, such as PAPLAL, ascorbic acid, and N-acetylcysteine, can improve *Sod1* loss-induced organ pathologies.

### PAPLAL normalizes gene expression, including that related to matrix biosynthesis and inflammation, in the skin

As shown in Figure 4, *Sod1* loss induced the transcriptional downregulation of *Col1a1* and *Has2*, as well as the upregulation of *Mmp2* expression, which were indicative of collagen and hyaluronic acid malformation in the atrophic skin of the *Sod1* ^−/−^ mice. *Sod1* loss also upregulated the expression of proinflammatory genes, such as *Tnf-*α and *Il-6*, in the skin (Figure 4). Tumor necrosis factor (TNF)- α regulates type I collagen expression via the c-Jun N-terminal kinase (JNK) and nuclear factor kappa-light-chain-enhancer of activated B cells (NF-κB) pathways in skin fibroblasts [34]. In addition, Galera *et al.* reported that NF-κB directly suppresses *COL1A1* gene transcription in human dermal fibroblasts [35] and accumulates in the nuclei of aged human fibroblasts in association with the downregulation of the *COL1A1* gene [36]. An IκB kinase (IKK)-β inhibitor has also been shown to suppress interleukin (IL)-1β-induced collagen degradation by inhibiting the activation of NF-κB and upregulation of matrix metalloproteinases [37]. Taken together, the inflammatory response controls collagen homeostasis via transcriptional mechanisms in fibroblasts. In the present study, PAPLAL treatment normalized the gene expression of *Col1a1* and *Tnf-α* in the skin of the *Sod1* ^−/−^ mice (Figure 4). With respect to other organs, Onizawa *et al.* reported that the intranasal administration of Pt nanoparticles reduced NF-κB activity and inhibited pulmonary inflammation in mice exposed to cigarette smoke [38]. Rehman *et al.* also reported that Pt nanoparticles have anti-inflammatory effects on the lipopolysaccharide-induced inflammatory response by downregulating the expression of IL-1β, TNF-α, and IL-6 in macrophages [39]. Collectively, these findings suggest that PAPLAL and Pt nanoparticles suppress the inflammatory response, resulting in improvements in the anabolic and catabolic regulation of collagen homeostasis.

### Pd nanoparticles stabilize the antioxidant activity of Pt nanoparticles by preventing their oxidative deterioration

Noble metal nanoparticles, such as Pd, Pt, and gold, are considered to function as antioxidants by reducing catalysis [19]–[21]. A number of studies have reported that Pt nanoparticles exhibit a strong ability to scavenge ROS, including O_2^−^_ and H_2_O_2_ [22]–[24]. In a lifespan analysis of *C. elegans*, Kim *et al.* reported that Pt nanoparticles extended the lifespans of wild-type N2 and short-lived *mev-1* nematodes, in which intracellular ROS accumulated due to respiratory impairment [40]. The present results demonstrated that Pt nanoparticles, but not Pd nanoparticles, possess SOD and catalase activity (Figure 5), which is consistent with the above results. Okamoto *et al.* reported that Pt nanoparticles were oxidized to PtO by oxygen in the air, which resulted in an time-dependent increase in their ability to degrade ascorbic acid [41]. However, the co-incubation of Pd nanoparticles with Pt nanoparticles effectively prevented PtO formation via Pt nanoparticles oxidation [41]. Since Pd has a lower oxidation/reduction potential than Pt, they proposed that Pd reduces Pt^2+^ to Pt in solution [41]. In order to further investigate the ability of Pd nanoparticles to prevent the oxidative deterioration of Pt, we herein examined the SOD and catalase activity of Pt nanoparticles after storage- or rotation-induced oxidization in the presence of Pd nanoparticles (Figure 5). Predictably, the co-storage of Pt nanoparticles with Pd nanoparticles delayed the oxidative inactivation of the SOD and catalase activity of the former nanoparticles, while in the absence of Pd nanoparticles the Pt nanoparticles were inactivated during incubation or rotation (Figure 6). Indeed, Pt nanoparticles that had been oxidized in air failed to suppress intracellular O_2^−^_ generation in *Sod1* ^−/−^ cells (Figure S2). In contrast, even after oxidation in air, PAPLAL, a mixture of Pd and Pt nanoparticles, continued to exhibit SOD and catalase activity, and hence, was able to decrease O_2^−^_ generation in *Sod1* ^−/−^ fibroblasts (Figure S2).

Notably, when Pd nanoparticles were added to Pt nanoparticles at a molar ratio of 3 or 4 to 1, the SOD and catalase activity levels of the Pt nanoparticles were sustained more effectively than when a molar ratio of 1 or 2 to 1 was employed (Figure 7). In fact, PAPAPL contains Pd and Pt nanoparticles at a molar ratio of 2.74 to 1, suggesting that the excess Pd nanoparticles in PAPLAL effectively protect the Pt nanoparticles from oxidative deterioration. Since long-term storage accelerates the oxidative deterioration of antioxidants in clinical use, the addition of Pd nanoparticles to such antioxidants might efficiently maintain their bioactivity under oxidative conditions.

### PAPLAL is a valuable antioxidant for delaying skin aging

In the present study, we directly applied PAPLAL to the skin of *Sod1* ^−/−^ mice. PAPLAL treatment effectively improved the skin thinning seen in the *Sod1* ^−/−^ mice, suggesting that the PAPLAL had been incorporated into epidermal and dermal cells. In a recently electron microscopic study, Okamoto *et al.* reported that the Pd and Pt nanoparticles in PAPLAL measure 3.59±0.56 and 1.93±0.34 nm, respectively, in diameter [41]. A previous study found that cells that had been treated with Pt nanoparticles exhibited increased Pt concentrations compared with the control cells [24]. In an inductively coupled plasma mass spectrometry-based study of *C. elegans*, Sakaue *et al.* reported that treatment with Pt nanoparticles increased the internalization of Pt in nematodes [42]. These results suggest that PAPLAL and/or Pt nanoparticles are transdermally taken in by cells, which inhibits the progression of skin pathologies caused by oxidative stress in mice.

In the present study, PAPLAL treatment did not cause any morphological abnormalities, such as cell infiltration or PAPLAL deposition, or cellular damage in mouse skin (Figures 2, 3). Furthermore, PAPLAL has been shown to be free from adverse effects during its clinical use as a treatment for chronic disease in humans [26]. In agreement with our results, several reports have found that treatment with Pt nanoparticles did not induce any alterations in the biological profiles of wild-type mice [38], [43]. On the other hand, Newkirk *et al.* recently reported that the oral administration of a mixture of Pd, Pt, and rhodium had a synergistic toxic effect on eosinophils in rats [44]. Further research on the dynamic state and safety of PAPLAL in the living body is required.

Vitiligo is an acquired depigmentation disorder characterized by H_2_O_2_/peroxynitrite-mediated oxidative and nitrative stress in the skin. Salem *et al.* reported that treatment with PC-KUS, a UVB-activated pseudocatalase, reduces the levels of epidermal H_2_O_2_ and induces repigmentation in vitiligo patients [45], [46]. Since PAPLAL possesses catalase activity, it might also be useful as a treatment for H_2_O_2_-related skin diseases, including vitiligo.

In conclusion, PAPLAL, which is composed of Pd and Pt nanoparticles at a molar ratio of 2.74 to 1, exhibits potent antioxidant activity (attributed to the effects of Pt nanoparticles) and attenuates aging-related skin pathologies *in vivo*. The Pd nanoparticles contained in PAPLAL prevent the oxidative deterioration of the Pt nanoparticles by attenuating PtO formation. PAPLAL has been found to have few adverse effects on skin morphology in transdermally treated mice. Consistent with these results, no previous studies have found that PAPLAL induces adverse effects, despite it being used in Japan to treat patients with chronic conditions for over 60 years. Therefore, PAPLAL is considered to be a safe and valuable antioxidant for delaying skin aging in humans.

## Supporting Information

Figure S1 **PAPLAL improves wound healing in aged mice.** (A) Typical pictures of aged C57BL/6 male mice that were treated with or without PAPLAL at six days after wounding. (B) Aged C57BL/6 male mice (17 months of age) were wounded on day 0 and treated with or without PAPLAL for 12 days. Wound size was measured over time. Data are shown as the mean ± SD; *p<0.05.
(TIF)

Figure S2 **PAPLAL suppresses O_2^−^_ production in** ***Sod1*** **-deficient fibroblasts.** *Sod1* ^−/−^ dermal fibroblasts were treated with 10 µM of Pt nanoparticles, 10 µM of Pd nanoparticles, or PAPLAL for 16 hours. Intracellular superoxide generation was detected using a DHE fluorescent probe and calculated as the area of DHE-based fluorescence divided by the number of Hoechst-positive cells. Data are shown as the mean ± SD. *p<0.05, **p<0.01.
(TIF)

Table S1 **qRT-PCR primers**
(DOCX)

Methods S1 **Supporting Methods.**
(DOCX)
