# Supplementary figures and images for: Palladium and Platinum Nanoparticles Attenuate Aging-Like Skin Atrophy via Antioxidant Activity in Mice
Source: PLoS One. 2014 Oct 15;9(10):e109288. doi: 10.1371/journal.pone.0109288 (PMC4198089; doi:10.1371/journal.pone.0109288)

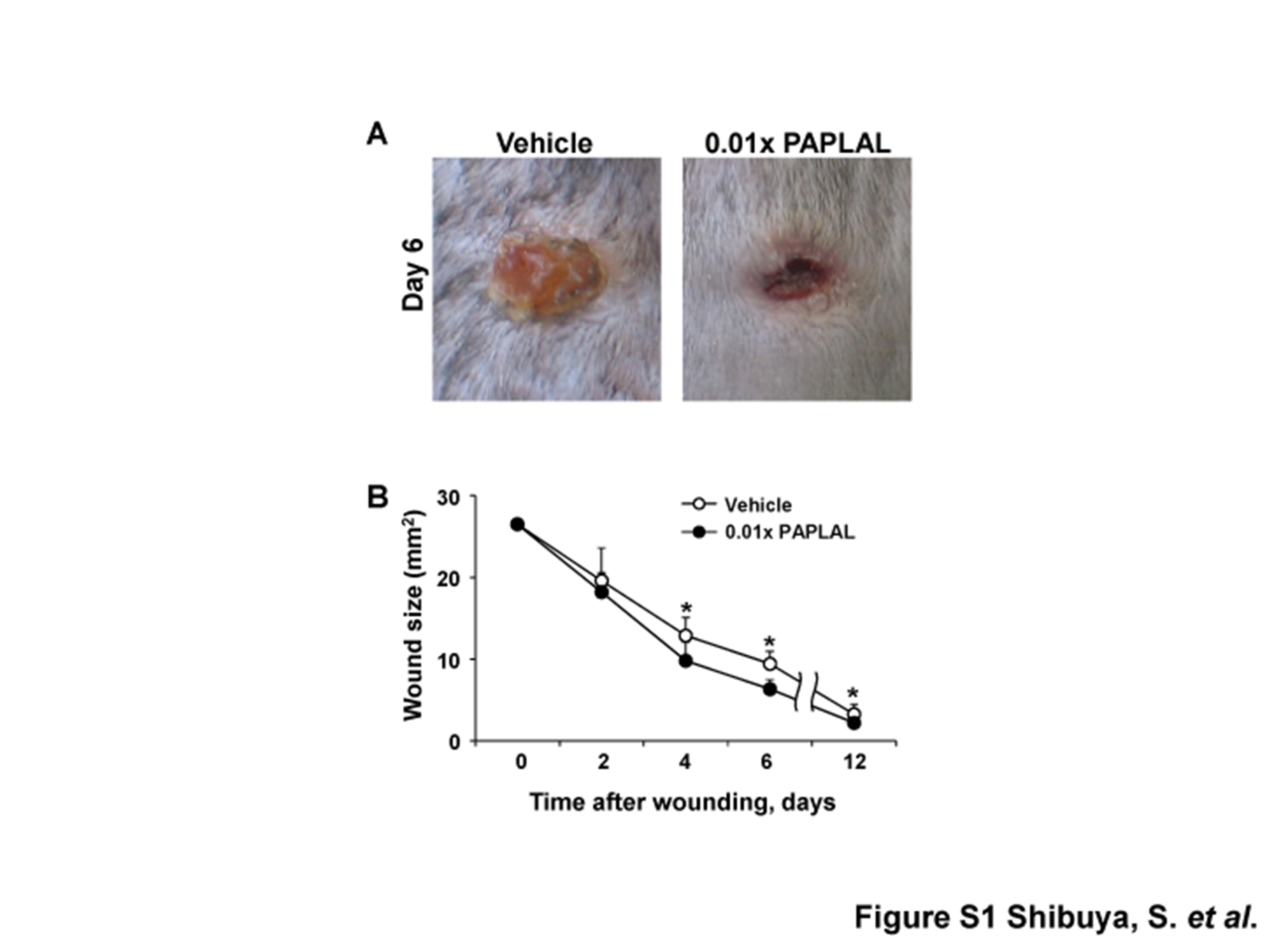

Supplement: Figure S1 — PAPLAL improves wound healing in aged mice. (A) Typical pictures of aged C57BL/6 male mice that were treated with or without PAPLAL at six days after wounding. (B) Aged C57BL/6 male mice (17 months of age) were wounded on day 0 and treated with or without PAPLAL for 12 days. Wound size was measured over time. Data are shown as the mean ± SD; *p<0.05. (TIF) [file pone.0109288.s001.tif]

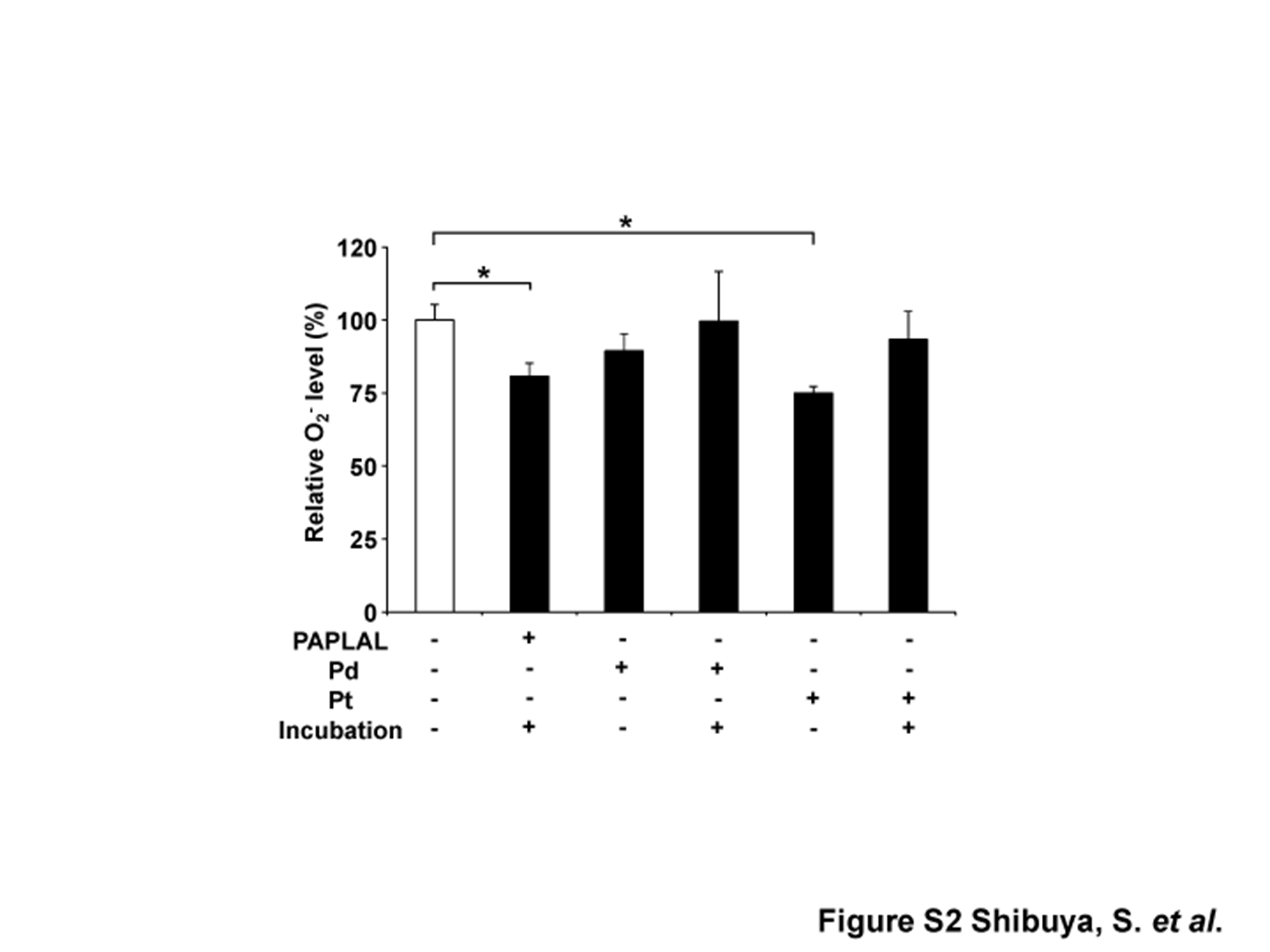

Supplement: Figure S2 — PAPLAL suppresses O2− production in Sod1 -deficient fibroblasts. Sod1 −/− dermal fibroblasts were treated with 10 µM of Pt nanoparticles, 10 µM of Pd nanoparticles, or PAPLAL for 16 hours. Intracellular superoxide generation was detected using a DHE fluorescent probe and calculated as the area of DHE-based fluorescence divided by the number of Hoechst-positive cells. Data are shown as the mean ± SD. *p<0.05, **p<0.01. (TIF) [file pone.0109288.s002.tif]
