# Supplementary material for: Palladium and Platinum Nanoparticles Attenuate Aging-Like Skin Atrophy via Antioxidant Activity in Mice
Source: PLoS One. 2014 Oct 15;9(10):e109288. doi: 10.1371/journal.pone.0109288 (PMC4198089; doi:10.1371/journal.pone.0109288)
Supplement: Table S1 — qRT-PCR primers (DOCX) [file pone.0109288.s003.docx]

**Table S1: qRT-PCR primers**

| **Gene Name** | **Forward** | **Reverse** |
| --- | --- | --- |
| *Col1a1* | CATGTTCAGCTTTGTGGACCT | GCAGCTGACTTCAGGGATGT |
| *Decorin* | TGATGCACCCAGCCTGAAAG | TCCATAACGGTGATGCTGTTGAA |
| *Gapdh* | AGAAGGTGGTGAAGCAGGCATC | CGAAGGTGGAAGAGTGGGAGTTG |
| *Has2* | CGGTCGTCTCAAATTCATCTG | ACAATGCATCTTGTTCAGCTC |
| *Il-6* | GCTACCAAACTGGATATAATCAGGA | CCAGGTAGCTATGGTACTCCAGAA |
| *Ki67* | GCTGTCCTCAAGACAATCATCA | GGCGTTATCCCAGGAGACT |
| *Mdm2* | CCCGAGTTTCTCTGTGAAGG | TCCTTCAGATCACTCCCACC |
| *Mmp2* | TAACCTGGATGCCGTCGT | TTCAGGTAATAAGCACCCTTGAA |
| *p53* | ACGCTTCTCCGAAGACTGG | AGGGAGCTCGAGGCTGATA |
| *Tnf-α* | ATGAGCACAGAAAGCATGATCCGC | GCTTGGTGGTTTGCTACGAC |
