# Supplementary material for: Palladium and Platinum Nanoparticles Attenuate Aging-Like Skin Atrophy via Antioxidant Activity in Mice
Source: PLoS One. 2014 Oct 15;9(10):e109288. doi: 10.1371/journal.pone.0109288 (PMC4198089; doi:10.1371/journal.pone.0109288)
Supplement: Methods S1 — Supporting Methods. (DOCX) [file pone.0109288.s004.docx]

**Supporting Methods**

**Wound healing model using mouse skin**

C57BL/6NCrSlc mice were purchased from Japan SLC (Shizuoka, Japan). The hair on the backs of aged C57BL/6NCrSlc male mice (17 months of age) was removed using electric clippers, and full-thickness wounds were created using a biopsy punch (6 mm in diameter). Then, the wound site was topically treated with PAPLAL or MilliQ water once a day for 12 days. Wound area was traced on a clear sheet and measured using the Qwin V3 imaging software.

**Cell culture**

Skin specimens were obtained from *Sod1*^-/-^ neonates at four to six days after birth, sterilized with 70% ethanol, and dissected into small pieces. Primary dermal fibroblasts were isolated via dissociation in 0.2% collagenase type 2 (Worthington Biochemical Corporation, Lakewood, NJ, USA) at 37°C for 60 minutes. The cells were maintained in α-MEM (Life Technologies Corporation) supplemented with 20% FBS (Life Technologies Corporation), 100 units/ml of penicillin (Sigma-Aldrich), and 0.1 mg/ml of streptomycin (Sigma-Aldrich) at 37°C in a humidified incubator under 5% CO_2_ and 1% O_2_ to prevent damage by O_2_. During the experiments, the cells were cultured under 20% O_2_ conditions.

**Assessment of intracellular superoxide generation**

*Sod1*^+/+^ and *Sod1*^-/-^ dermal fibroblasts were incubated under 1% O_2_ for one to two days, before being incubated under 20% O_2_ for eight hours to induce oxidative stress. The fibroblasts were continuously incubated with nanoparticles or vehicle for 16 hours in 20% O_2_. Then, the fibroblasts were incubated with a dihydroethidium (DHE) fluorescent probe (Invitrogen) (10 µM) and Hoechst 33342 (10 µM) (Calbiochem, Germany) for an additional 30 minutes under 20% O_2_. Intracellular O_2_^-^ generation and nuclei were detected using fluorescence microscopy (Leica). The area of the regions exhibiting DHE-based fluorescence and that of the regions exhibiting Hoechst 33342-based fluorescence (as an indicator of the number of cells) were measured using the Leica QWin V3 imaging software (Leica), and the level of intracellular O_2_^-^ generation was calculated as the area of DHE-based fluorescence divided by the number of Hoechst-positive cells.
